# Supplementary material for: Direction‐Dependent Conduction Polarity in Altermagnetic CrSb
Source: Adv Sci (Weinh). 2025 May 8;12(27):2502226. doi: 10.1002/advs.202502226 (PMC12279225; doi:10.1002/advs.202502226)
Supplement: Supplementary file 1 — Supporting Information [file ADVS-12-2502226-s001.pdf]

ADVANCED SCIENCE
Open Access

Supporting Information

for *Adv. Sci.*, DOI 10.1002/advs.202502226

Direction-Dependent Conduction Polarity in Altermagnetic CrSb

Banik Rai, Krishnendu Patra, Satyabrata Bera, Sk Kalimuddin, Kakan Deb, Mintu Mondal, Priya Mahadevan and Nitesh Kumar**

Supporting Information

Direction-Dependent Conduction Polarity in Altermagnetic CrSb

Banik Rai[1,3], Krishnendu Patra[1,3], Satyabrata Bera[2], Sk Kalimuddin[2], Kakan Deb[1], Mintu Mondal[2], Priya Mahadevan[1], and Nitesh Kumar[1]

[1]Department of Condensed Matter and Materials Physics, S. N. Bose National Centre for Basic Sciences, Salt Lake City, Kolkata-700106, India
[2]School of Physical Sciences, Indian Association for the Cultivation of Science, Jadavpur, Kolkata-700032, India
[3]These authors contribted equally.

1 EDXS and XRD

Figure S1 shows the typical EDXS spectrum of the elemental composition of CrSb and $Cr_{0.98}V_{0.02}Sb$. The EDXS results, obtained from several spots on single crystals of CrSb and $Cr_{0.98}V_{0.02}Sb$, are tabulated in Table S1. The elemental ratio for CrSb is close to 1:1. Upon doping with V, the nominal EDXS composition is nearly $Cr_{0.98}V_{0.02}Sb$.

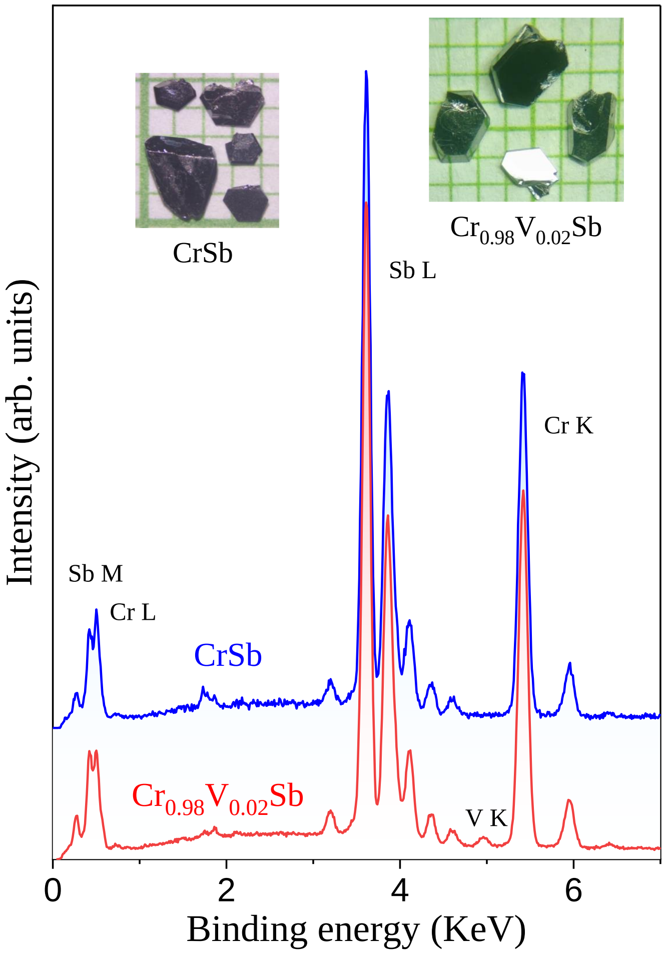

Figure S1. EDXS spectrum of CrSb (blue) and $Cr_{0.98}V_{0.02}Sb$ (red).

Table S1: Chemical composition and derived chemical formulas.

Compound	%Cr	%V	%Sb	$\langle\%Cr\rangle$	$\langle\%V\rangle$	$\langle\%Sb\rangle$	Chemical Formula
CrSb	49.1	-	50.1	50.325	-	49.675	$Cr_{1.0065}Sb_{0.9935}$
	50.3	-	49.7				
	51.0	-	49.0				
	50.9	-	49.1				
$Cr_{0.98}V_{0.02}Sb$	49.5	0.8	49.7	49.26	0.84	49.86	$Cr_{0.985(6)}V_{0.017(5)}Sb_{0.997(2)}$
	49.1	1.0	49.9				
	49.6	0.5	49.9				
	48.8	1.2	49.9				
	49.3	0.7	49.9				

Figure S2(a) shows the powder XRD pattern of CrSb fitted with the space group $P63/mmc$ using the Le-bail method. Fig. S2(b) shows the XRD pattern of a plate-like single crystal of CrSb. The observation of only $(000l)$ peaks confirms that the surface exposed to the XRD beam is ab-plane and the absence of other peaks indicates good crystallinity of the single crystal.

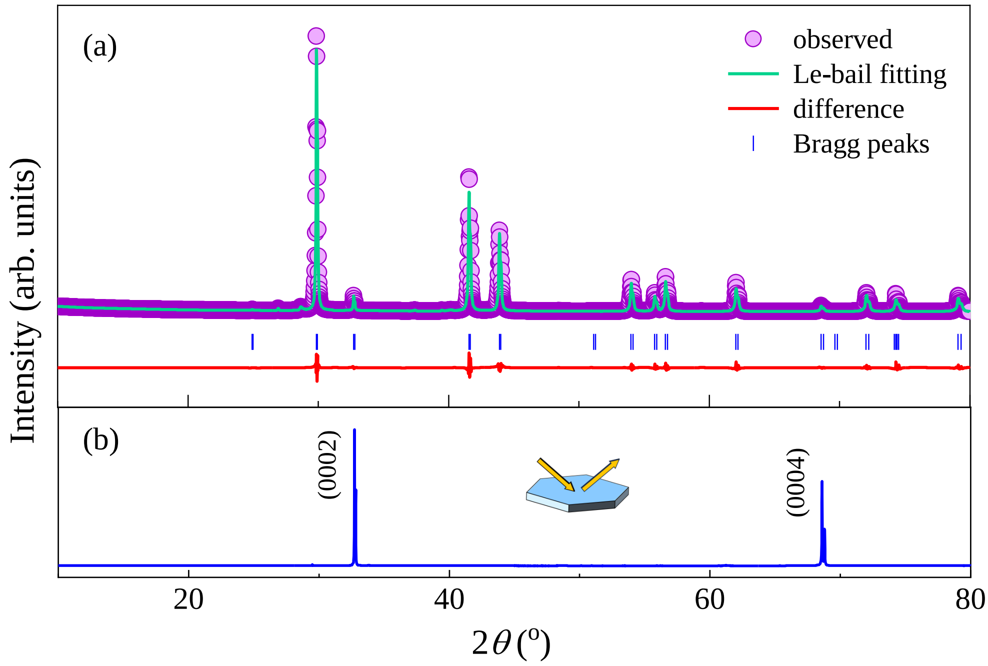

Figure S2. XRD study on CrSb. (a) Powder XRD pattern of CrSb fitted with space group $P6_3/mmc$ using the Le-bail method. (b) XRD pattern of a plate-like single crystal of CrSb.

2 Magnetoresistance and multi-carrier fitting

Figure S3(a)-(c) shows the magnetic field-dependent transverse magnetoresistance $\left[\mathrm{MR} = \frac{\rho_{ii}(B) - \rho_{ii}(0)}{\rho_{ii}(0)} \times 100\,\%\right]$ of CrSb at different temperatures, measured along the three orthogonal x, y, and z directions. MR reaches its maximum value of 8% for $B||$z [Figure S3(c)] at 9 T and 2 K. For the other two directions, the MR is smaller, with the smallest value observed for $B||$x [Fig. S3(a)]. In all directions, the observed positive MR can be attributed to the orbital cyclotron motion of charge carriers under the applied magnetic field. In systems with nearly equal electron and hole carrier densities (charge compensation), the MR typically exhibits a quadratic dependence on the magnetic field. [1] Figure S3(d), showing the MR at 2 K for all three directions, highlights the deviation of the MR from the quadratic behavior, indicating the presence of uncompensated charge carriers in CrSb.

In materials with multiple bands crossing the Fermi level (E_{F}), the mobility (μ) of charge carriers often varies between the bands. As a result, the charge carriers move independently within these bands, each

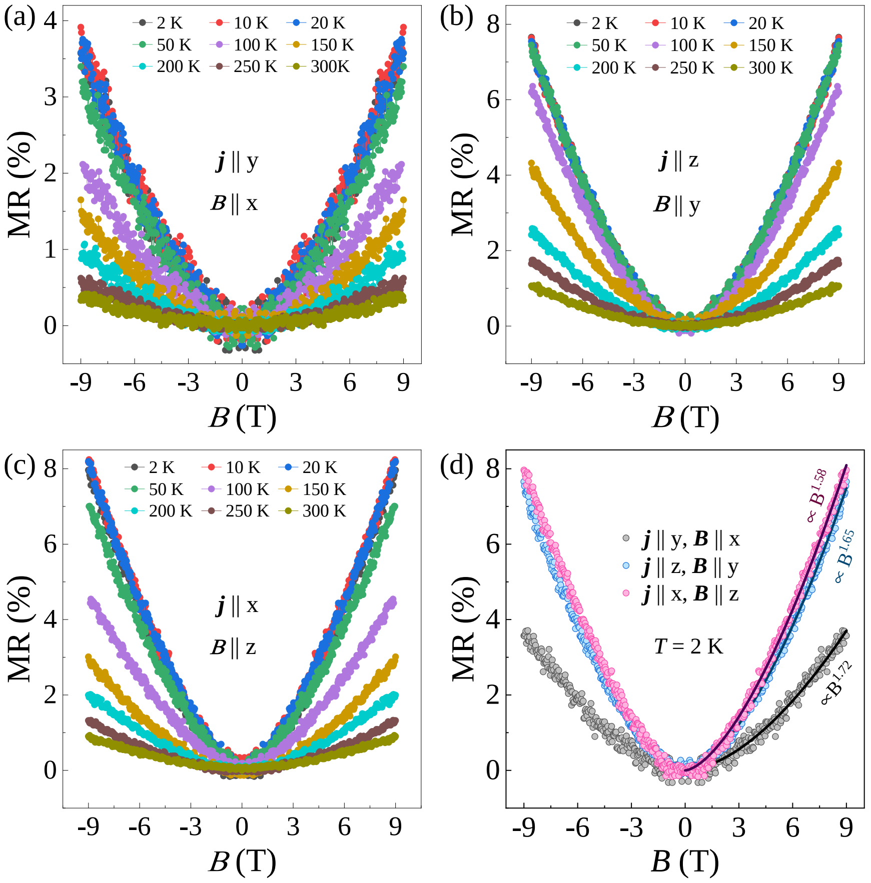

Figure S3. (a)-(c) Magnetoresistance of CrSb measured in various geometries at different temperatures. (d) Magnetoresistance at 2K measured along various directions and the corresponding exponential fit.

characterized by its own carrier density (n_i) and mobility (μ_i). [2] This results in a non-linear Hall signal as a function of the magnetic field. The longitudinal (σ_{xx}) and Hall (σ_{xy}) conductivity, considering the multi-carrier effect, can be expressed as:

$$\sigma_{\mathrm{xx}} = e\sum \frac{n_i\mu_i}{1+\mu_i^2B^2} \tag{1}$$

$$\sigma_{\mathrm{xy}} = eB\sum \frac{n_i\mu_i^2}{1+\mu_i^2B^2}. \tag{2}$$

Figure S4(a)-(b) shows the field-dependent σ_{xx} and σ_{xy} at 2 K fitted using equation (1) and (2), considering two and three carrier models. The fitting quality for two-carrier model is poor, indicating the presence of more than two independent types of charge carriers. The fitting improves significantly with the three-carrier model. This model also fits the data at higher temperatures, as shown in Figure S4(c)-(d). The values of n and μ obtained from the fit are presented in Figures S4(e) and S4(f), respectively. Of the three independent charge carriers, one carrier type is hole-like (h) and the other two are electron-like ($e1$

Table S2: Carrier density and mobility at various temperatures obtained by three carrier fit.

Temperature (K)	n_{h} ($10^{21}\mathrm{cm}^{-3}$)	n_{e1} ($10^{20}\mathrm{cm}^{-3}$)	n_{e2} ($10^{19}\mathrm{cm}^{-3}$)	μ_{h} ($\mathrm{cm^2V^{-1}s^{-1}}$)	μ_{e1} ($\mathrm{cm^2V^{-1}s^{-1}}$)	μ_{e2} ($\mathrm{cm^2V^{-1}s^{-1}}$)
2	1.45	6.39	1.75	312	480	2394
10	1.42	6.61	1.84	316	471	2344
20	1.39	6.78	1.87	315	458	2304
50	1.44	6.20	1.30	264	435	2196
100	1.12	7.63	0.89	204	281	1674
150	0.69	11.2	0.69	186	165	1293

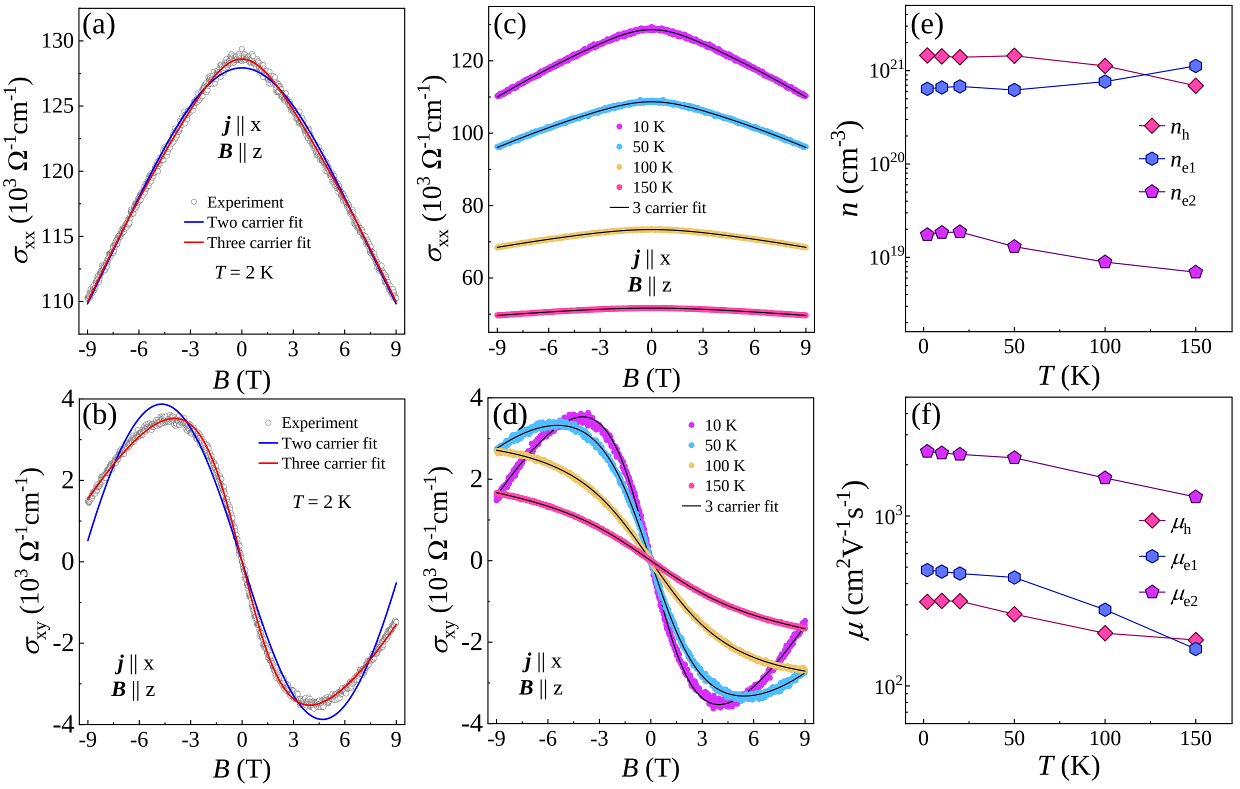

Figure S4. Multicarrier effect in the transport of CrSb. (a)-(b) Global (simultaneous) fitting of longitudinal ($\sigma_{\rm xx}$) and Hall ($\sigma_{\rm xy}$) conductivity at 2 K using two-carrier and three-carrier model. (c)-(d) Global fitting of $\sigma_{\rm xx}$ and $\sigma_{\rm xy}$ at various temperatures using three-carrier model. Variation of (e) carrier density and (f) mobility with temperature as obtained from three carrier fit. These values are in close agreement with the values reported in Ref. [3].

and $e2$). The hole-like carrier has the highest carrier density ($n_h \approx 1 \times 10^{21}\text{cm}^{-3}$). The two electron-like carriers have smaller carrier density, with one being much smaller than the other. The mobilities follow the opposite trend. The hole mobility (μ_h) is the smallest and the mobility of the second electron-like carrier (μ_{e2}) is the largest, reaching a value as large as 2394 $\text{cm}^2\text{V}^{-1}\text{s}^{-1}$ at 2 K. Table S2 shows the variation of carrier density and mobility of three carriers with temperature as obtained from the three-carrier fit.

The Fermi surface (FS) of CrSb consists of three pockets, namely α, β, and γ, corresponding to three distinct bands crossing $E_{\rm F}$ (see main text). Effective mass calculations (main text Table 1) reveal that the α and β are hole pockets, while γ is an electron pocket. This seemingly contradicts the results of the three-carrier fitting, which identifies two electron-like and one hole-like carriers. However, this apparent discrepancy can be resolved by considering the origin of the α and β pockets. These pockets arise from the altermagnetic and spin-orbit splitting of the same energy band. Consequently, they exhibit strong similarities in their electronic properties. Furthermore, the effective masses of the charge carriers residing within these pockets are nearly identical, suggesting that their mobilities are also highly comparable. Due to these strong similarities, the charge carriers within the α and β pockets do not behave as entirely independent entities in transport. Their contributions tend to be highly correlated, effectively merging into a single, dominant hole-like carrier contribution in the three-carrier fit. The first electron-like carrier ($e1$) in the three-carrier fit can be attributed to the electron pocket γ. The second electron-like carrier ($e2$) most likely correspond to the small hemispherical sub-pockets present within the α and β pockets, as these sub-pockets are electron-like in nature (see main text). Since, these sub-pockets are well separated from the larger portion of the α (or β) pocket and exhibit distinct geometry, the charge carriers residing on them can contribute independently to the transport, even though they arise from the same energy band. The value of n_{e2} is about 100 times smaller than n_h, as evident from Table S2. A similar proportion is visibly evident in the size of the larger portion of the α (or β) pocket and its sub-pocket. Since, carrier density is proportional to the volume of the corresponding Fermi pocket, our attribution of second electron-like carrier to the hemispherical sub-pockets is well supported. The value of n_{e1} is also consistent with the relative size of the γ pocket. In Figure S4, different carrier types, as obtained from the three-carrier fit, have been attributed to specific portions of the FS.

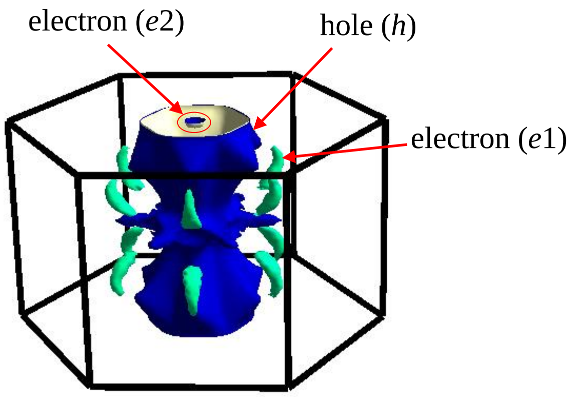

Figure S5. The attribution of different carrier types, as obtained from the three-carrier fit, to different portions of the FS.

3 Altermagnetic splitting of energy bands

Altermagnets have been identified as a new phase of collinear magnets that share properties with both ferromagnets (e.g., non-relativistic spin splitting of energy bands) and antiferromagnets (e.g., zero net magnetization) [4]. In altermagnets, the spatial arrangement of non-magnetic atoms surrounding the magnetic ones plays a crucial role in dictating their distinct spin group symmetry, which distinguishes them from ferromagnets and conventional antiferromagnets. Unlike conventional antiferromagnets, where the opposite spin sublattices are connected by a simple lattice translation or inversion, in altermagnets, this connection is established by a real-space crystal rotation. The symmetries of an antiferromagnetic crystal, i.e. $\mathcal{T}$, $\boldsymbol{t}\mathcal{T}$ and $\mathcal{PT}$, where $\mathcal{T}$, $\boldsymbol{t}$ and $\mathcal{P}$ are time reversal, lattice translation and space inversion operations, are thus broken in an altermagnet which facilitates the momentum dependent spin splitting of energy bands even without spin-orbit coupling akin to ferromagnets. For CrSb, the sublattice transposing symmetries are $C_{6z}t_{1/2}\tau$ and $M_z\tau$ (see main text). The first symmetry, $C_{6z}t_{1/2}\tau$, results in three spin-degenerate nodal planes in the Brillouin zone (BZ) as shown by the blue vertical planes in Figure S6(a). The second symmetry, $M_z\tau$, result in one spin-degenerate nodal plane in the BZ, as shown by a orange horizontal plane in Figure S6(a). Spin-degeneracy is lifted when moving away from these four nodal planes. Figure S6(b) shows the band structure of CrSb calculated along the path $\mathrm{L_1} - \Gamma - \mathrm{L_2}$ of the BZ in the absence of spin-orbit coupling (SOC). As this path lies outside the four nodal-planes, the bands experience significant splitting.

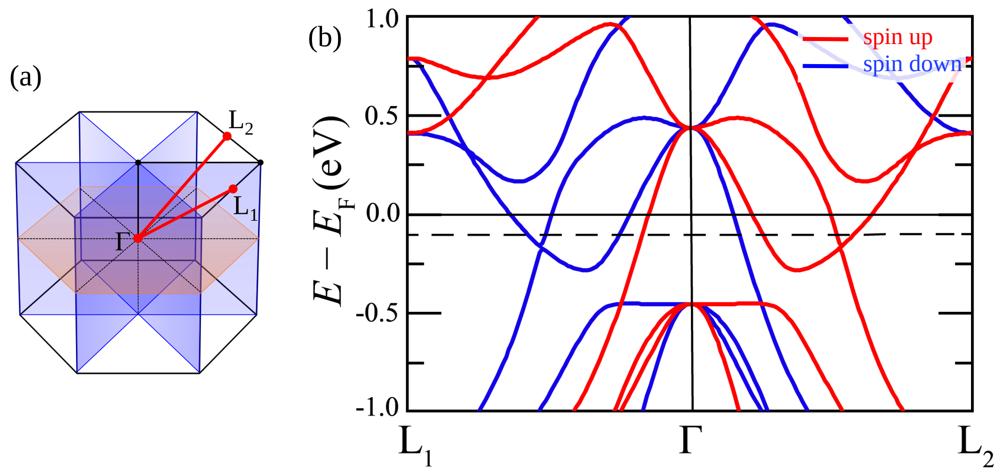

Figure S6. (a) Brillouin zone (BZ) of CrSb showing four spin-degenerate nodal planes. (b) Band structure of CrSb calculated in the absence of spin-orbit coupling (SOC) along the paths as shown in Figure S6(a). A significant altermagnetic splitting can be observed. The dashed horizontal line represents the experimental fermi energy (E'_{F}) (see main text).

4 Magnetic component-projected relativistic band dispersion

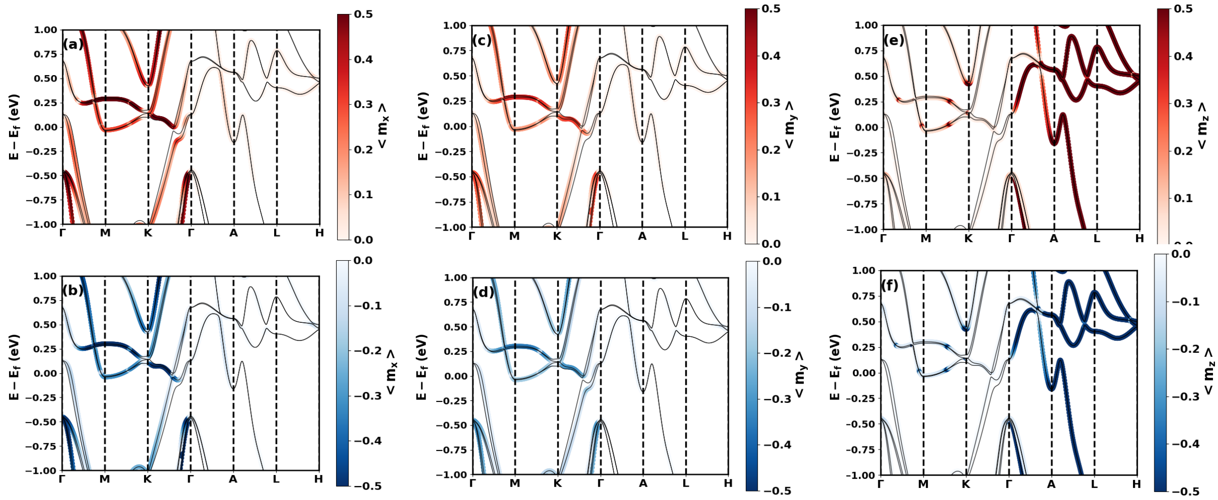

Figure S7. Band dispersions for CrSb plotted along various symmetry directions. Spin-orbit interactions have been included in the calculation of the band structure. The spin projections for positive (a) $\langle m_x \rangle$ (c) $\langle m_y \rangle$ and (e) $\langle m_z \rangle$ as well as negative (b) $\langle m_x \rangle$ (d) $\langle m_y \rangle$ and (f) $\langle m_z \rangle$ have been shown. The color bar on the right of each plot gives the magnitude.

5 Band dispersion at finite k_z plane

It is evident from the FS that there is no γ pocket at the $k_z = 0$ plane, as there is no intersection between band #3 and the shifted Fermi energy (E'_{F}) (indicated by dashed horizontal line) in that plane. However, at a finite k_z, band #3 intersects E'_{F} which gives rise to the γ pocket in the FS. In Figure S7, relativistic band dispersion is shown for the $k_z = 0.2\pi/c$ plane, where the band #3 intersects E'_{F}.

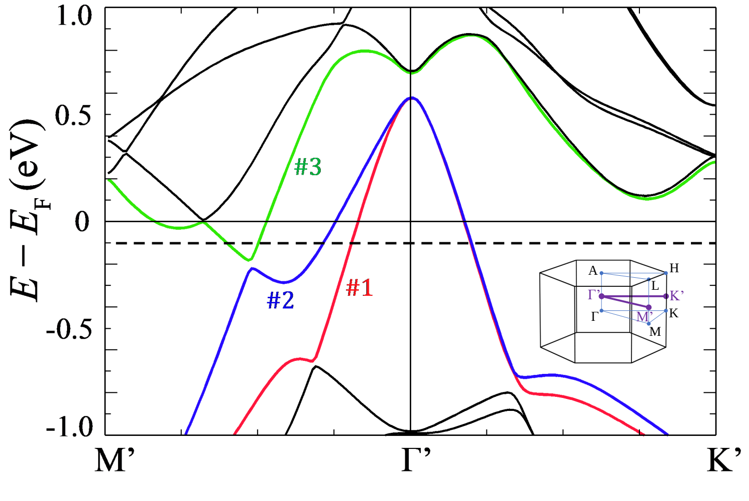

Figure S8. Band dispersion (with SOC turned on) along high symmetry lines for a particular fixed plane $k_z = 0.2\pi/c$.

6 Number of electrons per unit cell (u.c.) as a function of energy.

We have calculated the number of electrons at a particular energy using DFT calculations. From this, we determined the change in the number of electrons at the shifted Fermi energy (E'_{F}) as shown in Figure S9.

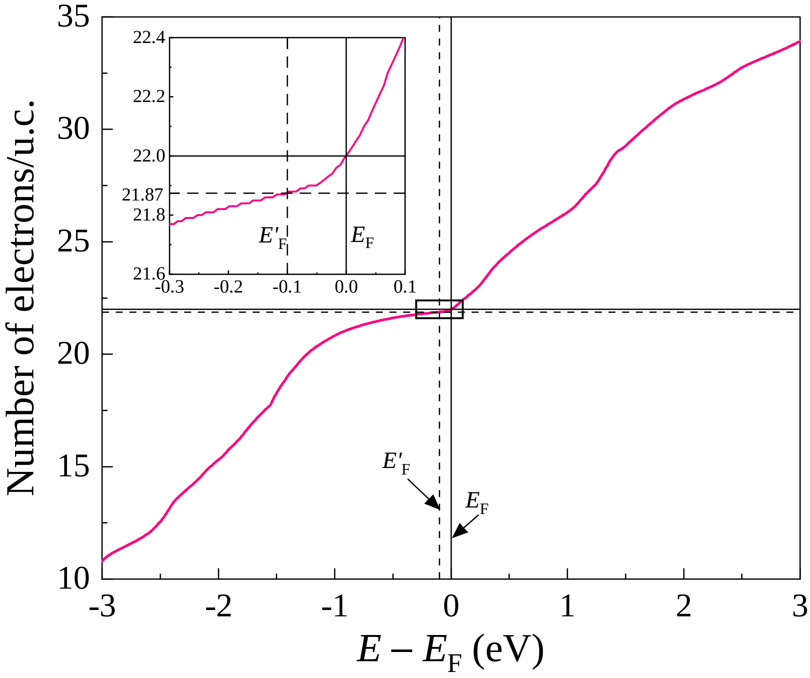

Figure S9. Number of electrons per unit cell as a function of energy.

7 Seebeck coefficient of $Cr_{0.98}V_{0.02}Sb$

To prove that a small amount of hole doping in CrSb destroy the DDCP, we have measured the in-plane Seebeck coefficient (S_{xx}) of $Cr_{0.98}V_{0.02}Sb$. CrSb is n-type in the *ab*-plane and thus has negative S_{xx}. In Figure S10, we show that S_{xx} changes sign and becomes positive for $Cr_{0.98}V_{0.02}Sb$, thus destroying the DDCP.

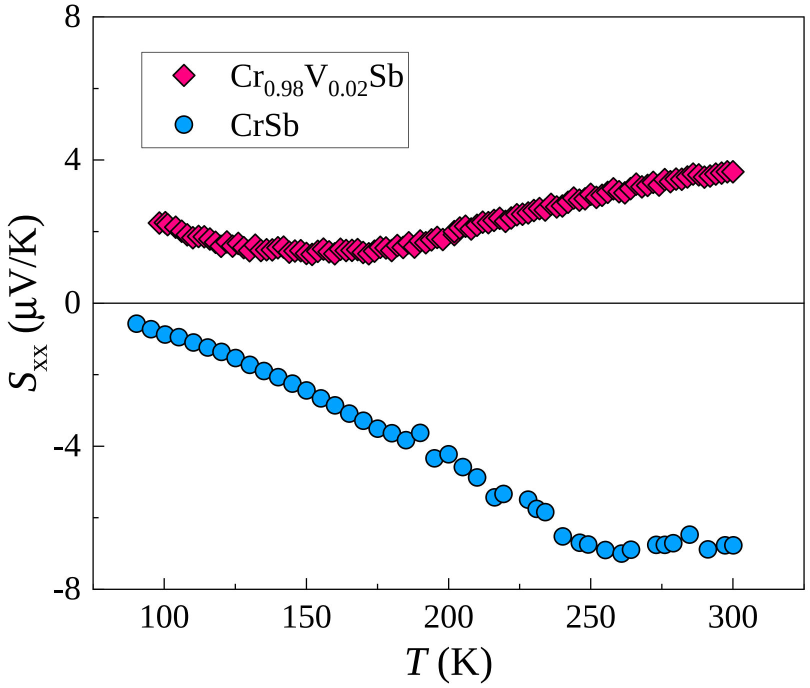

Figure S10. In-plane seebeck coefficient (S_{xx}) for CrSb and $Cr_{0.98}V_{0.02}Sb$, showing that the sign of S_{xx} has changed from negative to positive in $Cr_{0.98}V_{0.02}Sb$ because of hole (vanadium) doping.

References

[1] Neil W Ashcroft, N David Mermin, and Sergio Rodriguez. Solid state physics. *Am. J. Phys.*, 46(1):116–117, 1978.

[2] Colin Hurd. *The Hall effect in metals and alloys.* Springer Science & Business Media, 2012.

[3] Yuqing Bai, Xinji Xiang, Shuang Pan, Shichao Zhang, Haifeng Chen, Xi Chen, Zhida Han, Guizhou Xu, and Feng Xu. Nonlinear field dependence of hall effect and high-mobility multi-carrier transport in an altermagnet crsb. *App. Phys. Lett.*, 126(4), 2025.

[4] Libor Šmejkal, Jairo Sinova, and Tomas Jungwirth. Emerging research landscape of altermagnetism. *Phys. Rev. X*, 12(4):040501, 2022.
